# Findings and Lessons Learned From Strengthening the Provision of Voluntary Long-Acting Reversible Contraceptives With Postabortion Care in Guinea

**DOI:** 10.9745/GHSP-D-18-00344

**Published:** 2019-08-22

**Authors:** Anne Pfitzer, Yolande Hyjazi, Bethany Arnold, Jacqueline Aribot, Reeti D. Hobson, Tsigue G. Pleah, Shani Turke, Benita O’Colmain, Sharon Arscott-Mills

**Affiliations:** a Jhpiego, Washington, DC, USA.; b Jhpiego, Conakry, Guinea.; c Jhpiego, Baltimore, MD, USA.; d ICF, Rockville, MD, USA.; e Bill & Melinda Gates Institute for Population and Reproductive Health, Department of Population, Family, and Reproductive Health, Johns Hopkins Bloomberg School of Public Health, Abidjan, Côte d’Ivoire.; f ICF, Phoenix, AZ, USA.; g ICF, Lansing, NC, USA.

## Abstract

Integrating voluntary family planning into postabortion care (PAC) presents a critical opportunity to reduce future unintended pregnancies. Although Guinea has low contraceptive prevalence overall, acceptance of long-acting reversible contraceptives (LARCs) among PAC clients is higher than among interval LARC users and higher than the national average. In 2014, we assessed the extent of LARC provision within PAC services and the factors influencing integration. Primary and secondary data collected from 143 interviews, 75 provider assessments, and facility inventories and service statistics from all 38 public facilities providing PAC in Guinea allowed exploration of voluntary family planning uptake in the context of PAC. Study findings showed that 38 of 456 (8.3%) public health facilities or 38 of 122 (31.1%) facilities with a mandate to manage obstetric complications provided PAC services. Service statistics from 4,544 PAC clients in 2013 indicate that 95.2% received counseling and 73.0% voluntarily left the facility with contraception, with 29.6% of acceptors choosing a LARC. Family planning within PAC was emphasized in advocacy, policy and guidelines, quality improvement, and supervision, and the range of contraceptive options for postabortion clients was expanded to enable them to avoid a second unintended pregnancy. Factors that influenced provision of family planning within PAC included (1) the ability of champions both within and outside the Ministry of Public Health to advocate for PAC and leverage donor resources, (2) the incorporation of PAC with postabortion family planning into national policies, standards, and guidelines, (3) training of large numbers of providers in PAC and LARCs, and (4) integration of LARCs within PAC into quality improvement and supervision tools and performance standards. Guinea has gradually scaled up provision of PAC services nationwide and its experience may offer learning opportunities for other countries; however, continued advocacy for further expansion to more rural areas of the country and among private health facilities is necessary.

## Abstract (precis)

Integrating voluntary long-acting reversible contraceptive (LARC) methods within postabortion care (PAC) in Guinea has increased LARC uptake among PAC clients, compared with non-PAC clients. With aid from government champions and leveraging of resources, Guinea has incorporated PAC into national policies and guidelines and trained providers on PAC and LARCs to expand service provision.

## INTRODUCTION

Unintended pregnancy and induced abortion indicate an unmet need for contraception and suggest missed opportunities of family planning programs to serve clients. If a health facility manages a case with abortion complications without offering a full range of voluntary, highly effective contraceptive methods to prevent future unintended pregnancies, the health system has failed to provide comprehensive, client-centered care. Effective delivery of postabortion care (PAC) leads to decreased abortion-related maternal mortality and prevents repeat abortions.^1^ In 1994, at the International Conference on Population and Development, PAC emerged as a new public health intervention to improve management of abortion complications and prevent unintended pregnancies after an abortion.^2^^–^^5^ Since then, experts have introduced and expanded PAC service delivery to ensure that women receive judgment-free, compassionate care that includes (1) treatment of incomplete abortion using manual vacuum aspiration (MVA) when clinically indicated; (2) family planning counseling and provision of voluntary contraceptives before discharge, as well as referral to other reproductive health services; and (3) engagement with communities to reduce care-seeking stigma.^6^ Despite these efforts, however, few countries currently implement PAC at scale.^7^ Even where PAC is provided, some elements of the model, particularly family planning counseling and service provision, are poorly implemented.^4^^,^^8^^,^^9^ The exploration of effective models of PAC operating at scale in a country, especially in severely resource-constrained settings, can inform other countries.

**Figure:**
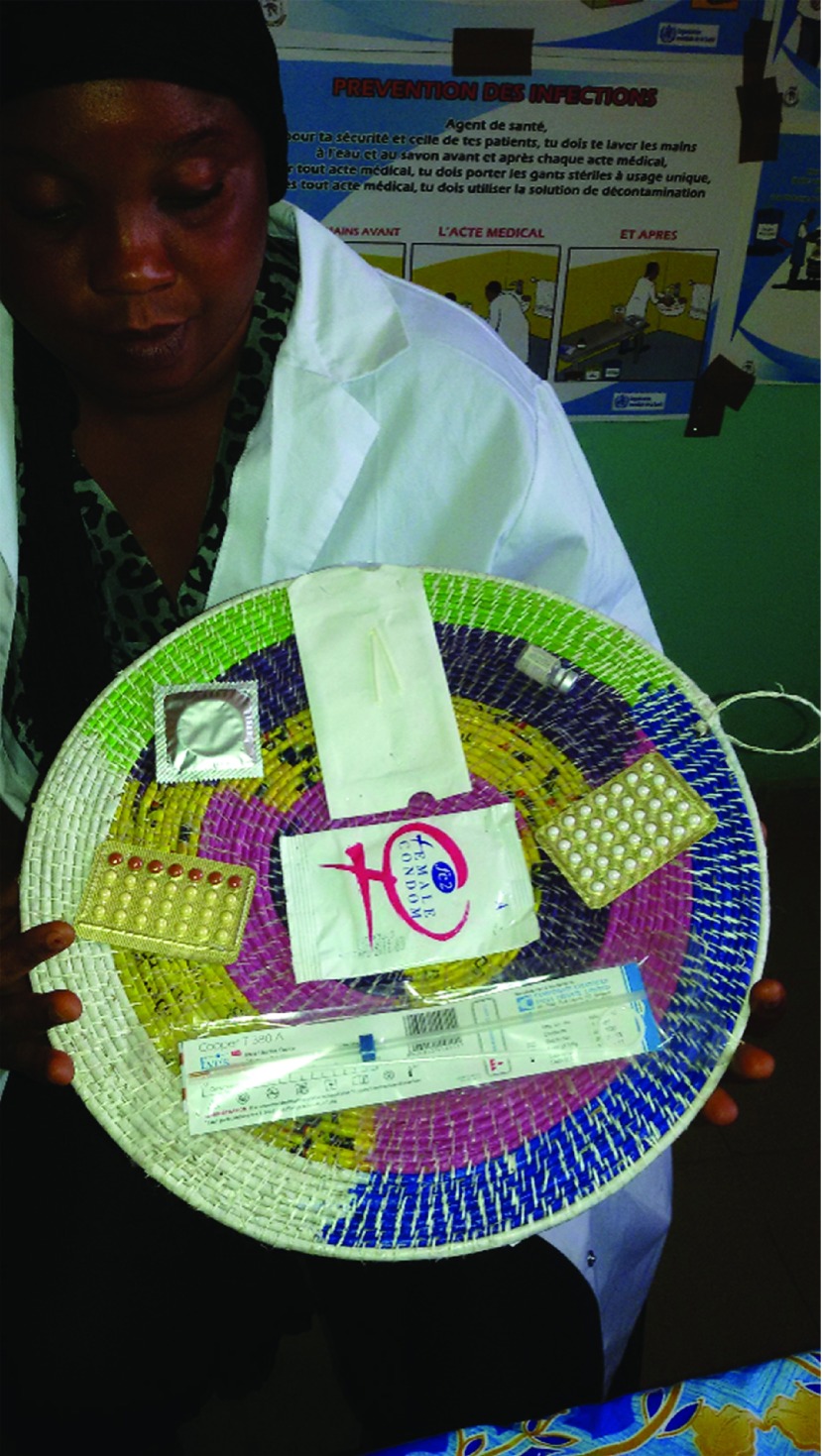
Contraceptive methods typically used in Guinea. © Suzanne Austin/Jhpiego

In studies exploring ways to improve PAC, large percentages of women accept voluntary family planning methods.^10^ Family planning experts have recently advocated for more tier-based counseling based on the contraceptive effectiveness of various methods with typical use of the method.^11^^,^^12^ A postabortion service interaction offers an opportunity for initiating a highly effective contraceptive method. This opportunity is often lost if a full range of methods is not available in the same location as the management of abortion complications. Recent papers have shared data on voluntary acceptance of long-acting reversible contraceptives (LARCs)^13^ or proportion of facilities that offer LARCs within PAC services.^14^

In many countries, implementation of comprehensive PAC services is complicated by the fact that services are provided by maternity staff. Historically, this staff may not have been trained or expected to provide LARCs or other contraceptive methods and thus tends to refer PAC clients to the family planning unit or recommend short-acting methods, if providing any family planning counseling or services at all. Sizeable loss to follow-up typically occurs when such referrals are made.^15^ Acceptance and continuation rates are higher when services are integrated and provided at the treatment point^16^ and the rates are even higher when the provider receives supportive supervision after training.^17^

### The Guinean Context

Following the 1994 International Conference on Population and Development, the West African nation of Guinea sought to improve the availability and quality of its PAC services. Abortion is legally restricted except when the life of the mother is in danger or in cases of incest, rape, or fetal impairment.^18^ The Guinean context for PAC implementation is challenged by the country’s low overall contraceptive prevalence. In 2017, only 11% of all women of reproductive age were using a modern contraceptive method. Among those using contraception, 2.8% relied on the intrauterine device (IUD), 1.4% on implants, 2.8% on female sterilization, 15.5% on the Lactational Amenorrhea Method, 22.5% on pills and injectables, and 33.5% on condoms.^19^

The Ministry of Health’s *2008–2012 Family Planning Repositioning Strategic Plan* sought both to expand voluntary method choice, including LARCs, and to strengthen PAC.^20^ Guinea implementers recognized early the importance of ensuring that a full range of contraceptive methods, including LARCs, is integrated into and available at the PAC point of treatment.^15^ An update of the Repositioning Plan for 2013–2018 reiterated the same commitment to PAC.^21^ Table 1 describes the evolution of Guinea’s PAC program, from the pilot phase in 1998, through expansion, to consolidation.

Guinea implementers recognized early the importance of ensuring that a full range of contraceptive methods, including LARCs, is integrated into and available at the PAC point of treatment.

**TABLE 1.** Evolution of the Postabortion Care Program in Guinea, 1998–2014

Phase and Years of Implementation	Cumulative No. of Health Facilities	Program Description	Donor	Types and Specific Names of Health Facilities
Introduction 1998–2001	12	Pilot in 2 national hospitals (1998 and 1999), followed by 10 additional sites in 2001, in Conakry and Upper Guinea. Activities included advocacy to stakeholders; development of PAC policies, standards, and protocols; site needs assessments; training in infection prevention, counseling skills, and family planning, and in abortion complication management using MVA; provision of initial stocks of equipment and supplies; transfer of learning visits to support organization of services, placement of contraceptives in PAC procedure room, and linkage to other reproductive health services and supportive supervision; site-level all-staff orientations about PAC, which often included engaging local officials and radios to inform them of the services. Policy: The MOPH first recognized the need for PAC, then led the development and finalization of the PAC policy, standards, and protocols. Subsequently, MOPH adopted the PAC implementation approach used in the first 12 health facilities as the standard for PAC introduction to be used in scale-up. All subsequent implementing partners were asked to follow the same approach.	USAID	National hospitals: Donka and Ignace Deen Regional hospitals: Faranah, Kankan Prefectural hospitals/municipal medical centers: Dabola, Dinguiraye, Mandiana, Siguiri, Kissidougou, Kouroussa, Kerouane Urban health center: Banan Koro
Early expansion 2002–2005	22	Activities included training of trainers, who then supported implementation of the same activities as above in order to integrate PAC in 10 new sites; community engagement meetings linked with initiation of PAC services at new sites.	USAID, UNFPA, unknown for selected health facility	Prefectural hospitals/municipal medical centers: Matam, Ratoma, Flamboyant, Minière, Forecariah, Pita, Boke, Boffa, Fria, Dubreka
Expansion 2006–2009	38	Policy: When the MOPH revised national reproductive health policies, standards, and protocols, PAC standards and protocols were incorporated into that document, which was finalized in 2006. Activities included those listed above in 16 new sites as well as supervision and refresher training for the 22 sites previously integrated. A 2008 regional PAC meeting in Saly, Senegal, and hosted by CEFOREP was the catalyst for additional PAC program consolidation, including: Fostering Change Virtual Leadership Program targeting 4 West African countries, including Guinea (2009–2010) Advocacy, tool development, and initial implementation in 5 Conakry health facilities of a quality improvement methodology of SBM-R^22^ Commercial vendor established and approved by MOPH to resupply MVA equipment	UNFPA and USAID	Regional hospitals: N’zérékoré, Kindia, Mamou, Labe Prefectural hospitals/municipal medical centers: Télimélé, Lelouma, Coleah, Beyla, Sinko, Gueckedou, Macenta, Lola, Yomou, Coyah Urban health center: Télimélé, Lelouma/Leysare
Support to existing sites 2010–2014	38	Activities included follow-up and supportive supervision of PAC activities in health facilities in Conakry and Upper and Forest Guinea, training of PAC service providers in LARCs, and rollout of SBM-R at 28 sites. Revision of community health worker educational materials regarding bleeding during pregnancy and postabortion family planning. 2013 regional meeting in Saly, Senegal, hosted by E2A, and evaluation visits of Fostering Change Program Countries.^23^	USAID and UNFPA	
Post-assessment 2014-present	48	Ebola virus epidemic-related disruptions to the health system. In recovery and reconstruction phase, training of providers and support to additional 10 sites, including 6 in prefectures not previously covered and in 4 urban health centers.	USAID	Prefectural hospitals/municipal medical centers: Dalaba, Tougué, Mali, Koubia, Gaoual, Koundara Urban health center: Dabola, Dubreka, Manquepas, Boffa

Abbreviations: CEFOREP, Centre Régional de Formation, de Recherche et de Plaidoyer en Santé de la Reproduction; E2A, Evidence to Action; LARCs, long-acting reversible contraceptives; MOPH, Ministry of Public Health; MVA, manual vacuum aspiration; PAC, postabortion care; SBM-R, Standards-Based Management and Recognition; UNFPA, United Nations Population Fund; USAID, United States Agency for International Development.

Notes: Guinea is geographically divided into 8 regions, 38 prefectures or municipalities (equivalent to a district), and 410 sub-prefectures. Each sub-prefecture has a health center (urban or rural). Prefectural hospitals have an average catchment population of 304,804, and regional hospitals cover 1,447,819 population. Users pay fixed amounts defined by the Ministry of Health for each type of service and facility, thus PAC service costs are fixed at 6000 francs (US$0.82) in health centers, 10,000 francs ($1.37) in prefectural or regional hospitals, and 15,000 francs ($2.05) in teaching hospitals. This price does not include contraceptive services, which incur a small additional fee. Donors supply the great majority of contraceptives offered through government health facilities in Guinea.

A program report from 2013 indicated that family planning counseling was more common and uptake of contraceptives among PAC clients was higher in Guinea than in other West African countries.^23^ Further, contraceptive usage included considerable voluntary uptake of LARCs. Specifically, the report found that 100% of PAC clients in 3 Burkinabe facilities were counseled on family planning and 47% left with a method; 79% of PAC clients at 3 sites in Togo were counseled and all those counseled left with a method, mostly combined oral contraceptive pills; 17% of Senegal PAC clients at 2 sites were counseled and 4% left with a method; whereas, all PAC clients at 3 Guinea sites were counseled and left with a method, with a predominance of IUDs and injectables. Healy et al.^10^ found a range in acceptance among PAC clients within studies of 37% to 87% discharged with a contraceptive method. In Ethiopia, postabortion contraception uptake exceeded 58% of PAC clients.^24^ Due to higher effectiveness and continuation, LARCs are generally more successful in preventing repeat unintended pregnancies and repeat abortions.^25^

Our study aimed to (1) determine the extent of success in implementing the 2006 PAC policy and 2008 Repositioning Plan, with emphasis on voluntary uptake of LARCs, within PAC services in Guinea, and (2) understand health systems factors influencing PAC policy implementation. Specifically, we conducted a census in early 2014 of public health facilities providing PAC services to determine the proportion that offered a full range of contraceptive methods, including LARCs, within PAC, and how elements of health systems functioning affect PAC and LARC integration within PAC.

## MATERIALS AND METHODS

**FIGURE 1:**
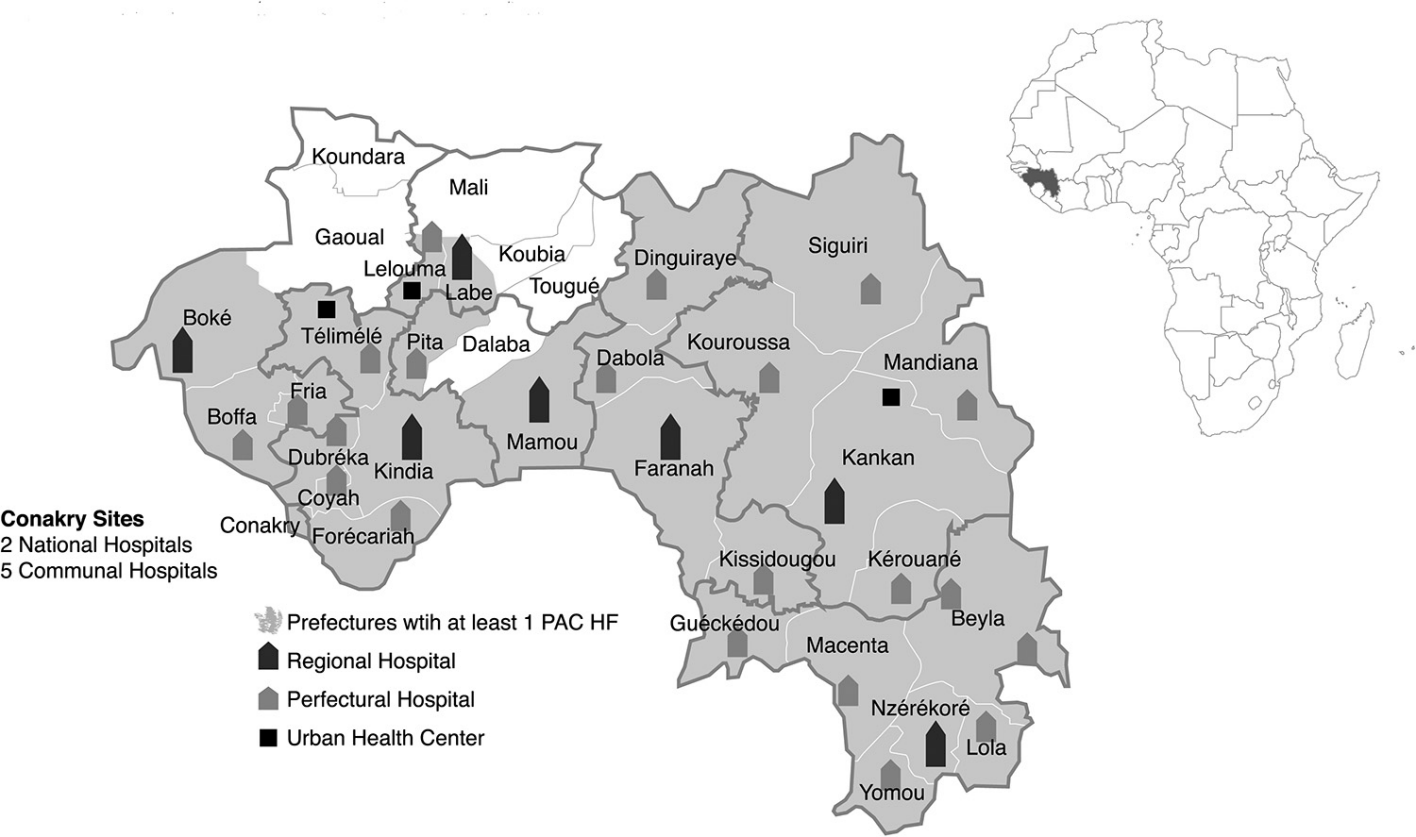
Location and Type of PAC Facilities in Guinea Abbreviations: HF, health facility; PAC, postabortion care.

We employed a cross-sectional observational design using both primary and secondary data sources. Data collectors visited all 38 public facilities that provide PAC in Guinea (Figure 1). Ten Guinean physicians and midwives with experience providing maternity and PAC services collected data during a 1-month period in early 2014. They did not collect data in facilities where they worked. Ethical approval was obtained from the Johns Hopkins Bloomberg School of Public Health Institutional Review Board and the Guinea National Ethical Committee for Health Research.

The study team developed interview and assessment tools from a list of domains around provision of voluntary family planning and LARCs within PAC and health systems factors to support expansion and sustainability of services (e.g., coverage of services, quality, characteristics of PAC facilities, support for provision of LARCs within PAC). We developed questionnaires for national stakeholders, prefecture focal points, maternity in-charges, and PAC providers. Interview tools included close-ended questions with precoded response options, with a few open-ended questions to gauge opinions on integration for all respondents. The national stakeholder questionnaire included more open-ended questions to facilitate a better understanding of the current policy environment and health systems factors contributing to the quality of PAC services and integration of LARCs. Table 2 lists the number of respondents sampled as well as a summary of the questions presented to each respondent. The supplemental material to this article details infection prevention and PAC equipment and supplies included in the inventory. Data collection tools used in PAC facilities were pretested in a recently renovated medical center attached to the Ministry of Social Affairs that was not included in the 38 facilities during data collector training.

**TABLE 2.** Characteristics of Respondents by Facility Type, Provider Qualification, and Study Tools Administered

	Prefectures/Municipal Focal Points^a^	Maternity In-Charges^b^	PAC Providers	Total Facilities
**Respondents^c^ by facility type**				
National hospitals	—	2	7	2
Regional hospitals	—	7	22	7
Prefectural hospitals	—	26	42	26
Urban or improved health centers	—	3	4	3
Total	—	38	75	38
**Respondents by qualification**				
Obstetrician/ gynecologist	—	10	5	15
General practitioner	16	23	23	62
Nurse/laboratory technician^d^	7	2	5	14
Midwife	3	—	23	26
Auxiliary nurse	4	3	18	25
Medical student	—	—	1	1
Total	30	38	75	143
**Study tool administered^e^**	Questions on lists of facilities in their respective jurisdictions, whether facilities offered PAC, commodity availability, financing for activities, supervision reporting, and plans for expansion	Questions on staffing by cadre overall and for PAC services, staff training, hours and organization, recordkeeping, and supervision	Questions on types of services available, availability of supplies, attitudes and opinions about PAC and contraception; assessment of provider knowledge and skills in postabortion family planning counseling	

Abbreviation: PAC, postabortion care.

^a^ Includes the head of the health office, the reproductive health focal point, and others who are responsible for more than one facility.

^b^ Includes medical officer in charge of maternity, head of PAC unit, and deputy/assistant maternity in-charge.

^c^ In addition to these subnational respondents, the study team interviewed 3 national stakeholders from the family health division.

^d^ Among PAC providers, 1 of the 5 nurses was a laboratory technician.

^e^ A separate facility assessment tool was also administered, which included an inventory of equipment and supplies and abstraction of service statistics from PAC registers.

National stakeholders and key informants at the prefectural level were selected based on their ability to provide information on PAC implementation in Guinea. Thus, prefectural level reproductive health focal points were interviewed instead of the heads of the prefectural health office. Within facilities, we employed a convenience sampling strategy to interview maternity in-charges and PAC providers present at the time of data collection. All facility-based respondents consented to be interviewed.

Data were collected on tablets and synchronized daily to a cloud-based server using the mobile data collection platform CommCare.^26^ Automatic constraints were programmed into study instruments to minimize human error during data entry and to reduce the data cleaning required following data collection. Data collectors reviewed the data and performed initial data cleaning; the study team conducted additional cleaning before analysis.

Descriptive statistics were tabulated for interviewee responses and facility assessment results. To address health systems-related factors affecting PAC, we analyzed responses from multiple respondents related to training, supervision, equipment and supplies, and the health management information system (HMIS). Because these data were collected a few years ago, we also present more recent updates in Table 1 and 2017 service statistics from the national health information system later in the discussion section.

## RESULTS

### Implementation of National Policy to Expand PAC Inclusive of Postabortion Contraception

Data collectors completed 143 interviews in all 30 prefectures/communes and 38 facilities (Table 2). At the time of the assessment, 38 (8.3%) of 456 health facilities provided PAC services in Guinea (Table 3). According to national policy, 122 of the 456 facilities were mandated to provide PAC, thus 31% of those mandated to provide PAC did so. All national hospitals provided PAC services except one, which was established in 2013 and generally did not offer any maternity services. All regional and three-quarters of prefectural hospitals and municipal medical centers provided PAC services. PAC provision remained minimal (3.8%) in urban health centers.

Of the facilities mandated to provide PAC, 31% did so.

**TABLE 3.** Number and Proportion of Health Facilities Offering Postabortion Care, by Level, Along With Parameters of Service Availability and Family Planning Integration, as Reported by Maternity In-Charge Respondents

Facility Type	Facilities in Country^a^	Facilities With PAC Services^b^	Facilities With PAC Services Available 24/7	Facilities With Any FP Services Provided in PAC Unit	Facilities With Both LARC Methods in PAC Unit^c^	Facilities With Both LARC Methods in FP Unit
	No.	No. (% of total health facilities)	No. (% of PAC health facilities)	No. (%)	No. (%)	No. (%)
National hospitals	3	2 (66.7)	2 (100.0)	2 (100.0)	2 (100.0)	2 (100.0)
Regional hospitals	7	7 (100.0)	7 (100.0)	7 (100.0)	6 (85.7)	7 (100.0)
Prefectural hospitals/municipal medical centers	34	26 (76.5)	26 (100.0)	26 (100.0)	25 (96.2)	26 (100.0)
Urban health centers	78	3 (3.8)	1 (33.0)	3 (100.0)	3 (100.0)	3 (100.0)
Rural health centers	334	0 (0.0)	—	—	—	—
Total	456	38 (8.3)	36 (94.7)	38 (100.0)	36 (94.7)	38 (100.0)

Abbreviations: FP, family planning; LARC, long-acting reversible contraceptive; PAC, postabortion care.

^a^ Based on Guinea National Health Management Information System.

^b^ One of the urban health centers (in the same town as a prefectural hospital) had PAC services in place in prior years but not during the whole of calendar year 2013, because of the lack of manual vacuum aspiration equipment.

^c^ The LARCs available in Guinea are Copper T380A intrauterine devices and Jadelle subdermal implants.

National stakeholders (3) reported the existence of national PAC strategies and the intention to expand services to the 6 prefectures where PAC services had not yet been introduced, despite challenges accessing these areas. They also reported dependence on donors and technical assistance both for expanding PAC and for supplying contraceptive commodities, especially for costlier implants.

Within facilities providing PAC, services were available 24/7 at nearly all facilities (94.7%, n=36). Use of MVA as a method of PAC management was reported among nearly all providers (99%). Six (8%) also reported using dilation and curettage, 8 (11%) reported using medical management, and 2 (3%) reported using all 3 methods. Availability of family planning is universal in the PAC unit, where abortion complications are managed. According to maternity in-charge reports, provision of voluntary LARCs within the unit was nearly universal (94.7%); however, 2 clarified that PAC clients seeking LARCs are referred to the family planning unit. While all maternity in-charges reported availability of LARC in PAC or family planning units, service statistics showed that 6 (17%) health facilities offered no LARCs to PAC clients in 2013—2 in Boke, 2 in Kindia, and 1 in both N’zérékoré and Labe.

**FIGURE 2.**
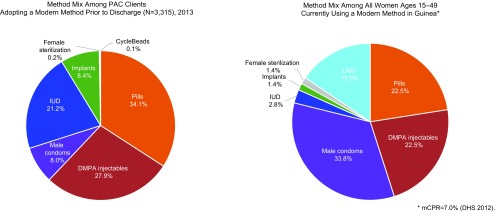
Contraceptive Method Mix Among Postabortion Clients Choosing a Method Prior to Discharge and Among All Women Ages 15–49 in Guinea Currently Using a Modern Method Abbreviations: DHS, Demographic and Health Survey; DMPA, depot medroxyprogesterone acetate; IUD, intrauterine device; LAM, Lactational Amenorrhea Method; mCPR, modern contraceptive prevalence rate; PAC, postabortion care.

Data from 2013 facility registers showed that among PAC cases (n=4,544), nearly all clients (95.2%) received counseling and nearly three-quarters (73.0%) voluntarily chose and left with a family planning method before discharge (Table 4). Among family planning acceptors, 29.6% chose a LARC method (Figure 2). In comparison, nationally only 7.0% of all women 15–49 years old were using a modern method, based on data from the 2012 Demographic and Health Survey (DHS). Among these modern method users, only 4.2% were using LARCs. Family planning counseling rates and method uptake among PAC clients varied by region (Table 4). FP counseling rates exceeded 95% in most regions, but were lower in Labe and Kindia. Method uptake varied from 31% to 94%.

**TABLE 4.** PAC Caseload, Family Planning Counseling, and Family Planning Use, Health Facility Register Data, 2013

**Region**	PAC Cases	PAC Cases Counseled on Family Planning	PAC Clients Leaving the Facility With a Method	PAC/Family Planning Clients Who Chose a LARC Method
	No.	No. (% of PAC cases)	No. (% of counseled PAC clients)	No. (% of family planning acceptors)
Conakry	2,215	2,209 (99.7)	1,775 (80.1)	623 (35.1)
Kindia^a^	471	360 (76.4)	231 (49.0)	44 (19.0)
Boke	148	141 (95.3)	84 (56.8)	5 (6.0)
Mamou	300	286 (95.3)	228 (76.0)	97 (42.5)
Labe	295	223 (75.6)	92 (31.2)	10 (10.9)
Faranah	205	199 (97.1)	133 (64.9)	19 (14.1)
Kankan	692	691 (99.9)	567 (81.9)	118 (20.8)
Nzérékoré	218	217 (99.5)	205 (94.0)	66 (32.2)
Total	4,544	4,326 (95.2)	3,315 (73.0)	982 (29.6)

Abbreviations: IUD, intrauterine device; LARC, long-acting reversible contraceptive; PAC, postabortion care.

^a^ Two health facilities in Kindia region, a health center and a prefectural hospital, did not have any service statistics data for 2013. The health center staff indicated that they referred all PAC cases to a nearby hospital (in same city).

### Health System Factors Linked With PAC Implementation

At time of data collection, a total of 276 health care workers provided PAC in Guinea (Table 5). Conakry had the highest number of PAC providers (1–39 staff per site). N’zérékoré had the fewest PAC providers (median of 3 providers per site). To date, 416 providers have been trained in PAC in Guinea; half received group-based PAC training and half learned on the job. After Conakry, Boke had the most trained providers, whereas Labe and Faranah had the fewest. Among providers trained in LARCs, more were trained in IUDs than in implants, but some regions had relative parity in the numbers of providers trained in both methods. Family planning training data are presented for all providers in the facility without disaggregating by those also trained in PAC, so we were unable to determine the proportion of PAC providers who also received family planning training.

**TABLE 5.** Health Systems Factors Influencing Effective FP/LARC Integration Within PAC

	Region								
	Conakry	Kindia	Boke	Mamou	Labe	Faranah	Kankan	Nzérékoré	Total
**Health systems factors**									
PAC health facilities (N)	7	6	3	2	3	4	6	7	38
No. of staff providing PAC services^a^	119	30	23	15	14	19	34	22	276
Median	8	5.5	9	7.5	6	5	5.5	3	5
Range	1–39	1–8	5–9	3–12	1–7	4–5	2–10	0–5	1–39
**Availability of trained personnel^b^**									
PAC (Total)	128	43	54	38	31	33	49	40	416
Group-based PAC course	70	22	28	19	11	16	27	18	211
On the job	58	21	26	19	20	17	22	22	205
FP/LARCs									
IUD	54	18	13	15	9	17	24	26	176
Implants (Jadelle)	23	16	12	15	9	12	16	17	120
**Supervision tools incorporate PAC inclusive of FP**									
Internal or external supervision or both	7	5	3	2	3	4	6	7	37
**Equipment and supplies available**									
IEC materials	6	5	1	2	3	4	5	6	32
Contraceptives in MVA room									
Condoms	5	5	2	0	2	3	2	3	22
Pills (POP/COC)	5	5	3	1	2	4	5	6	31
DMPA	5	4	2	0	1	2	4	6	24
IUD	6	4	3	2	3	4	4	5	32
Implants	6	6	3	2	3	3	5	5	32
MVA equipment and supplies	3	0	1	0	0	0	0	3	7
Infection prevention supplies^c^	4	1	1	0	3	0	3	7	19
No. (%) of health facilities with any stock-outs of contraceptives	1 (14)	2 (33)	2 (67)	0 (0)	2 (67)	2 (50)	4 (67)	4 (57)	17 (45)
No. (%) of health facilities with a complete set of minimum equipment and supplies	3 (43)	0 (0)	0 (0)	0 (0)	0 (0)	0 (0)	0 (0)	2 (29)	5 (13)
**HMIS functioning**									
No. (%) of health facilities that report PAC data^d^	6 (86)	5 (83)	3 (100)	2 (100)	3 (100)	3 (75)	6 (100)	7 (100)	35 (92)

Abbreviations: COC, combined oral contraceptive; DMPA, depo-medroxyprogesterone acetate; FP, family planning; HMIS, health management information system; IEC, information, education, and communication; IUD, intrauterine device; LARC, long-acting reversible contraceptive; MVA, manual vacuum aspiration; PAC, postabortion care; POP, progestin-only pill.

Data sources include maternity in-charge responses and the facility assessments.

^a^ The investigators could not confirm the number of PAC providers in one health facility in N’zérékoré; thus providers are included from only 6 health facilities in that region.

^b^ We assume that maternity in-charges did not list the same providers as both trained through group-based and on-the-job PAC training.

^c^ Exhaustive list of 28 items that included access to water source, cleaning supplies, personal protective equipment, means to sterilize or high-level disinfect MVA equipment and instruments, containers, antiseptics, and disinfectants.

^d^ Two health facility maternity in-charges, one in Conakry and one in Kindia, did not report the PAC data they collected. One health facility in Faranah did not collect PAC data at all.

Among PAC cases in 2013, nearly all clients (95%) received family planning counseling and nearly three-quarters left the facility with a method.

Maternity in-charges and prefecture-level focal points were asked about health system factors that favor comprehensive PAC including contraceptive counseling and services, particularly LARCs. Nearly half (n=18) of the 38 facilities reported that their internal and external supervisory tools or standards included PAC and family planning (Table 5). National respondents confirm that PAC and family planning within PAC are captured in national supervisory tools.

Material availability was variable. Only 5 facilities (13%) had a complete set of basic information, education, and communication materials, as well as equipment and supplies. At the time of the assessment, most facilities (n=32) had LARCs available in MVA procedure rooms, although complete MVA supplies were found in only 7 facilities. Nineteen facilities had appropriate infection prevention supplies, with N’zérékoré reporting 100% availability across its facilities. Prefectural focal points in 5 of the 8 regions reported stock-outs of contraceptives in the last 6 months in 50% or more of PAC procedure rooms.

## DISCUSSION

Our study sought to assess the extent of PAC policy implementation in Guinea, with a focus on family planning and specifically LARC integration. At the time of the study in 2014, 31 of 38 prefectures had facilities offering PAC according to the international definition.^2^ PAC was predominantly available in hospitals in urban areas. This unequal distribution of services is not unique to PAC. The Guinea Costed Implementation Plan notes that although 62% of the population lives in rural areas, only 16% of service providers practice in those settings.^21^ However, within the Guinea health system pyramid of care, rural health centers are expected to manage only normal pregnancy and birth and systematically refer complications, including postabortion clients, to urban facilities. With the referral system taken into consideration, PAC facilities are intended to cover urban and rural zones in their catchment area. Thus, if the referral system is adequate to cover the needs of the population, the denominator for urban health centers and higher-level facilities totals 122 facilities. The coverage for PAC services at the time of the study was then 31.1% of facilities expected to deliver PAC services.

We found that 36 of 38 facilities offering PAC reported also offering a full range of contraceptives to their clients, and 32 actually did so in 2013. As a result, 73% of women left a facility with a contraceptive method after a spontaneous or induced abortion and 26% opted for LARCs, an encouraging trend given their particular efficacy and suitability in preventing repeat pregnancy.

The level of postabortion contraceptive uptake in Guinea compares favorably with levels reported elsewhere.^27^^,^^28^ Postabortion voluntary contraceptive adoption rates (73%) found in the 2013 service statistics stand in sharp contrast to the overall 7% rate of modern contraceptive method use among all women in Guinea in 2012 (or even the 11% estimate for today),^19^ perhaps indicating that the contraceptive demands of this high-need population are being met. Similarly, the uptake of IUDs after abortion at 21.2% among those adopting a method prior to discharge is in marked contrast to the 2.8% uptake of IUDs among modern method users of reproductive age nationally in 2012 .^29^ Our results demonstrate that PAC clients are more likely to use a contraceptive method after an abortion when counseled effectively and given a choice among a diverse array of methods. The results also suggest that Guinea was able to implement PAC policies that mandate comprehensive counseling and broad method choice. Even higher uptake might have occurred if these facilities had not had contraceptive stock-outs. The practice in Guinea of offering contraceptive methods in the same room as the management of postabortion complications may contribute to our findings, as also seen in Ghana.^30^ Low contraceptive prevalence overall does not appear to constrain postabortion contraceptive acceptance.

PAC clients are more likely to use a contraceptive method after an abortion when counseled effectively and given a choice among a diverse array of methods.

Another study objective was to understand how the Guinean health system has integrated and institutionalized PAC, inclusive of voluntary LARCs, including human resources, training, performance management through supervision, availability of essential equipment and supplies, and availability and use of PAC data. We found the highest numbers of PAC providers in the capital, consistent with the previously observed trend in both Guinea and elsewhere that health care workers and resources are often concentrated in urban areas or the nation’s capital, creating health care shortages in rural and more remote locations.^31^^–^^33^ While this can be partially explained by the inclusion of tertiary hospitals in Conakry, other facilities were also well staffed. In contrast, the Forest region had a low median number of PAC providers, likely due to its remoteness. Yet, despite lower staffing levels, the proportion of PAC clients counseled and the rate of family planning uptake were among the highest in the Forest region. Factors other than staffing levels, such as quality of counseling training, local leadership, champions, or donor-funded program support, may contribute to these results. However, the 2014–2015 Ebola outbreak undoubtedly worsened the situation, as other research has documented.^34^

Maternity in-charges were asked to give numbers of providers trained. Their reported number (416) far exceeds the number they reported as currently providing services (276). Transfers of staff to rural facilities that do not offer PAC could partially explain this. Conversely, the high proportion of providers they reported as trained on the job may explain how public facilities continue to offer PAC services integrated with family planning after external assistance ends. The ability of facilities to engage new providers in PAC service delivery is encouraging for continuing sustainability. Other interventions beyond training, such as the use of performance standards for PAC, may also contribute to higher quality service delivery. Furthermore, the offer of LARC training that likely included PAC providers is clearly important, although our inability to accurately determine how many providers received both types of training is a limitation.

Reports by maternity directors suggest that PAC is well integrated into existing supervision tools and standards, reinforcing the practice of comprehensive PAC. Standards in Guinea have multiple purposes. Beyond supervision, quality teams or individual providers use them as a job aid to improve the quality of their services. This suggests either that leadership is greater at the facility level or that the emphasis on PAC is a function of its integration into ongoing quality improvement tools.

The national health system was weakest in terms of the availability of equipment and supplies, HMIS analysis, and use of PAC data. Alarmingly, half of the facilities were missing essential infection prevention supplies and few had a full complement of MVA equipment.^†^ A challenge in Guinea is that each facility must procure its own MVA syringes and cannulas from a private commercial vendor. Our facility inventory assessment revealed the limitations of this system. These system challenges affecting consistency of key commodities, PAC supplies, and infection prevention supplies impede health care providers’ ability to deliver quality services and are not unique to Guinea. Shortages in health commodities in other sub-Saharan African countries have been well documented, and they contribute to ineffective provision of reproductive and maternal health care.^35^

System challenges affecting consistency of key commodities, PAC supplies, and infection prevention supplies impede health care providers’ ability to deliver quality services.

Only 8.3% of all Guinea health facilities, or 31.1% of those mandated, offered PAC services in 2014. The Guinea health system should further extend PAC service delivery closer to women. Guinea has already adopted task shifting of MVA for PAC to nurses and midwives, but more effort is needed to equip and support their performance of those competencies in rural health centers without easy access to referral facilities. Guinea stakeholders could consider expanding the options for managing incomplete abortion complications through use of misoprostol in rural health centers, as other countries have done,^36^ and emphasize all elements of PAC, including community mobilization. The Box reflects recent updates since the assessment, with expansion into 6 remaining prefectures that were not covered at the time of the study. One commune in Conakry continues to lack a PAC facility, and continued expansion will likely require donor support.

BOX **2018 Guinea Postabortion Care Situation Update**

Several years have passed since our data were collected. In addition, the assessment occurred prior to the Ebola virus crisis in Guinea. Therefore, we reviewed postabortion care (PAC) expansion and training that have occurred in the intervening years. As shown in Table 1, providers were trained in PAC, including long-acting reversible contraceptive (LARC) skills, at an additional 10 sites post-assessment, as well as at existing sites that needed training of replacement staff. Six of the 10 new sites are in the 6 prefectures that had no coverage in 2014. The remaining 4 were additional urban health centers in prefectures where PAC was available at the hospital level. Government policy remains to expand PAC to health centers equipped to offer basic emergency obstetric care. In 2018, these totaled 137, for a coverage of 35%.

The health management information system (HMIS) was revised in 2015 and now includes additional indicators, for example, disaggregation of PAC services by method and age group. New HMIS tools and reporting forms are gradually being rolled out, along with the use of District Health Information System (DHIS) 2.

A review of 2017 data collected by USAID partners reveal PAC cases to be below the 2013 level (3,260 in 2017 versus 4,544 in 2013). This decrease may be partially explained by facility usage having drastically declined during the 2014 and 2015 Ebola epidemic, and the slow recovery has meant that previous thresholds for maternal health have not yet been realized. Further, the temporary closure of both large high-volume national hospitals in Conakry for several months in 2017 led many clients to seek services in other health facilities, potentially including private structures that do not report their data to the national HMIS. The vast majority of cases in the HMIS were documented as receiving counseling for family planning (3,234 or 99.2%). In 2017, the acceptance of a family planning method after PAC services remained high at 76.7%, with even higher uptake of LARCs at 47.2%.

Our study provides a comprehensive picture of PAC service provision in Guinea. By interviewing multiple respondents at a facility, using tablets programmed for that purpose, and triangulating answers with service statistics and inventory data, we were able to cross-check responses by health facility (e.g., provision of voluntary LARCs to PAC clients in the past year in service statistics versus report from maternity in-charge). There are few published reports of national-level assessments of PAC services. One study in Afghanistan focused on assessing provider competence to manage abortion complications,^14^ while one in Ethiopia included both PAC and safe abortion services using signal functions for assessing facility readiness to manage even the most severe complications.^37^ Another study in Ethiopia described a baseline assessment that uncovered inadequate space within safe abortion and PAC procedure areas and shortages of commodities and supplies for LARC services as constraining the ability of facilities to offer integrated postabortion contraception.^38^ Recent studies have reported on subnational assessments in Ethiopia, Ghana, and India^17^^,^^30^^,^^38^ and shown increases in contraceptive uptake including voluntary LARCs and permanent methods.

**Figure:**
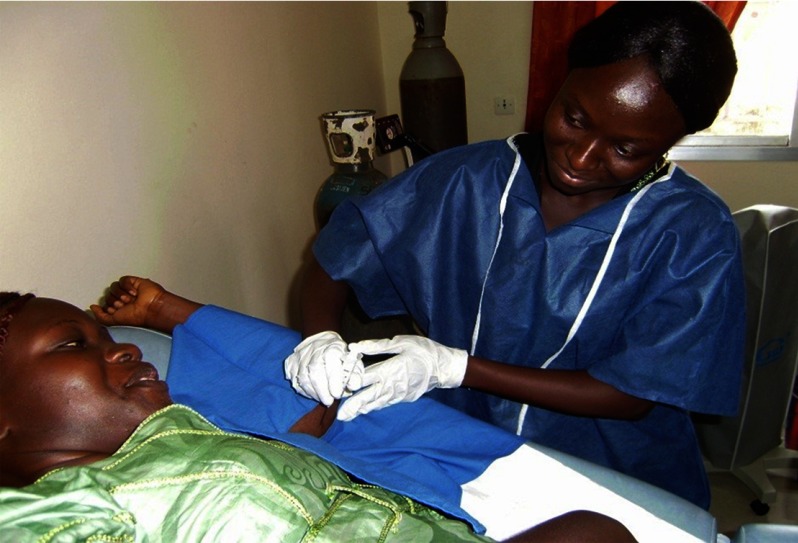
A client in Guinea chooses to use implants for contraceptive protection. © 2012 Suzanne Austin/Jhpiego

### Study Limitations

Because PAC and emergency obstetric care are linked and it is unknown whether Guinea’s health system offers adequate emergency obstetric care coverage, this study does not claim to understand met need for PAC or population-based coverage. Also, this study does not include private health facilities, which should be considered in future PAC program activities.

## CONCLUSION

We studied components of PAC services in a country that legally restricts abortion and where the rate of family planning uptake among married women is low. We found that all but one of the facilities expected to provide PAC were doing so, with a high proportion of clients receiving contraceptive counseling and voluntary family planning services within the PAC treatment unit. A relatively high proportion of women opted for LARCs in this setting, confirming the importance of providing voluntary, client-centered family planning services within the PAC treatment unit, including counseling and provision of voluntary LARCs as part of a comprehensive range of methods. More needs to be done to extend PAC to remote and private health facilities.

A relatively high proportion of women opted for LARCs in this setting, confirming the importance of providing voluntary, client-centered family planning services within the PAC treatment unit.

Factors that influence provision of family planning within PAC and expand the range of contraceptive options for postabortion clients include (1) the ability of champions both within and outside the Ministry of Public Health to advocate for PAC and leverage donor resources; (2) the inclusion of PAC with postabortion family planning into national policies, standards, and guidelines; and (3) training large numbers of providers in PAC and LARCs as well as the integration of tools and performance standards for quality improvement and supervision to encourage orienting providers new to a facility. Efforts subsequent to this study to incorporate additional PAC family planning indicators into the HMIS, coupled with better analysis of HMIS data, should further sustain PAC with high family planning adoption. Improvements in the contraceptive supply chain to avoid stock-outs remain urgently needed. Guinea’s experience with integrating and expanding voluntary family planning services, including LARCs, into PAC demonstrates that it is possible to achieve high family planning uptake among postabortion clients even when overall contraceptive use is low.

## Supplementary Material

supplemental material

## Footnotes

^†^USAID funds were not used to procure or distribute MVA equipment. USAID funds can be used to conduct trainings, monitoring, and assessments to understand facility level availability or other issues related to PAC.

## Notes

**Funding:** Funding for this study was made possible through support provided by U.S. Agency for International Development, Office of Population and Reproductive Health, USAID/Washington D.C., under the terms of Award No. GHS-A-00-08-00002-00 (Maternal and Child Health Integrated Program) Leader with Associates Cooperative Agreement. Manuscript development and printing were supported under the terms of the Cooperative Agreement AID-OAA-A-14-00028 (the Maternal and Child Survival Program).

**Peer Reviewed**
