# Supplementary material for: Findings and Lessons Learned From Strengthening the Provision of Voluntary Long-Acting Reversible Contraceptives With Postabortion Care in Guinea
Source: Glob Health Sci Pract. 2019 Aug 22;7(Suppl 2):S271–84. doi: 10.9745/GHSP-D-18-00344 (PMC6711623; doi:10.9745/GHSP-D-18-00344)
Supplement: supplemental material [file GHSP-D-18-00344_index.html]

Supplement to Findings and Lessons Learned From Strengthening the Provision of Voluntary Long-Acting Reversible Contraceptives With Postabortion Care in Guinea | Global Health: Science and Practice

## Supplemental material

**Files in this Data Supplement:**

- List of Manual Vacuum Aspiration (MVA) and Infection Prevention Supplies - Text s01, PDF
