# Supplementary material for: Findings and Lessons Learned From Strengthening the Provision of Voluntary Long-Acting Reversible Contraceptives With Postabortion Care in Guinea
Source: Glob Health Sci Pract. 2019 Aug 22;7(Suppl 2):S271–84. doi: 10.9745/GHSP-D-18-00344 (PMC6711623; doi:10.9745/GHSP-D-18-00344)
Supplement: supplemental material [file 18-00344-Pfitzer-Supplement.pdf]

Pftizer A, Hyjazi Y, Arnold B, et al. Inclusion of long-acting reversible contraceptives as an option for postabortion care clients in Guinea: results of an observational study. *Glob Health Sci Pract*. 2019;7(suppl 2). https://doi.org/10.9745/GHSP-D-18-00344

Supplement: List of Manual Vacuum Aspiration (MVA) and Infection Prevention Supplies

EQUIPMENT AND SUPPLIES FOR POSTABORTION CARE (PAC) SERVICE DELIVERY

In PAC Procedure Room

- Instrument tray
- Stethoscope
- Blood pressure cuff
- Thermometer
- Emergency kit (drugs and emergency materials)
- Supplies for blood testing
- Kidney dish
- 10-12 ml syringes with 22 gauge for paracervical block
- MVA kit: 2 complete kits with (double valve syringes, adaptor lubrication fluid, replacement rings, different sizes of cannulas 5 to 10 and 12)
- Complete set of instruments (2 tenaculums, 2 speculum, and 1 ring forceps)
- Light source

Additional Nice-to-Have Items

- Curettes – small, medium, and large
- Dilators Pratt (metal) or Denniston (plastic)

Supplies for IUD Service Delivery Immediately After MVA

- Uterine sound
- IUD in sterile package
- Speculum
- Scissors

PAC PROCEDURE ROOM DRUGS

Analgesics

- Aspirin or Ibuprofen
- Pethidine (or appropriate substitute)

Large-Spectrum Antibiotics

- Ampicillin
- Chloramphenicol
- Metronidazole (Flagyl)
- Sulfamethoxazole-trimethoprim (Cotrimoxazole)
- Tetracycline

INFECTION PREVENTION

Equipment and Supplies for Infection Prevention

- Goggles for provider
- Antiseptic solution
- Utility gloves
- Gauze/cotton
- Plastic bucket for decontamination solution
- Sharps container
- Leak-proof container for contaminated waste
- Storage container with cover
- Alcohol

Instrument Processing

- Heat source and pot/pressure cooker for high-level disinfection (boiling or steam)
- Autoclave (steam) or dry heat sterilizer
- Clean water to clean instruments or high-level disinfection
- Boiled water for rinsing instruments after chemical disinfection
- Distilled water for rinsing instruments sterilized chemically
- Soap
- Detergent
- Plastic containers
- Utility gloves
- Surgical gloves
- Exam gloves
- Personal protective equipment (aprons, masks, etc.)
- Small brushes

Antiseptics

- Iodine preparation (Provodon-iodine, Bétadine)
- Chlorhexidine, 4% (Hibitane, Hibiscrub)
- Disinfectants
- Sodium hypochlorite 3.5%
- Glutaraldehyde 2% (Cidex)

Uterotonics

- Misoprostol
- Oxytocin

Intravenous Solutions

- Ringer lactate
- Glucose, 5% and 50%

Pftizer A, Hyjazi Y, Arnold B, et al. Inclusion of long-acting reversible contraceptives as an option for postabortion care clients in Guinea: results of an observational study. *Glob Health Sci Pract*. 2019;7(suppl 2). https://doi.org/10.9745/GHSP-D-18-00344

- Potassium chloride
- Dextrose, 5%
- Normal saline
- Plasma substitute (Dextran or Heamacel)

Vaccinations and Injections

- Anti-tetanus
- Distilled water
